# The Surgeon’s Perspective: Promoting and Discouraging Factors for Choosing a Career in Surgery as Perceived by Surgeons

**DOI:** 10.1371/journal.pone.0102756

**Published:** 2014-07-15

**Authors:** Julia C. Seelandt, Reto M. Kaderli, Franziska Tschan, Adrian P. Businger

**Affiliations:** 1 Institute of Work Psychology, University of Neuchâtel, Neuchâtel, Switzerland; 2 Department of Visceral Surgery and Medicine, Inselspital University Hospital Bern, Bern, Switzerland; 3 Military Medical Service, Swiss Armed Forces, Ittigen-Bern, Switzerland; 4 Private University in the Principality of Liechtenstein, Triesen, Liechtenstein; University of Michigan, United States of America

## Abstract

**Background:**

The aim of this study was to identify the factors perceived by surgeons that promote surgery as an attractive or unattractive career choice for today’s graduates. In addition, it examined whether the perspectives of surgeons in different professional situations converges. The content of work, contextual work conditions, and calling to this job are discussed in the context of choosing surgery as a career.

**Methods:**

Eight hundred sixty-nine surgeons were asked to answer open-ended questions regarding the factors that promote surgery as an attractive or unattractive career choice for today’s graduates. Four hundred ninety-two surgeons participated, and 1,525 statements were analyzed using Mayring’s content-analyses method. Chi-square tests were used to analyze the differences among hierarchical positions.

**Results:**

With respect to the factors that promote surgery as a profession, 40.8% (209/492) of the surgeons stated that surgery is a calling, 29.1% (149/492) of the surgeons provided at least one argument related to the positive task characteristics, and 12.9% (66/492) of the surgeons provided statements related to the positive contextual factors. With respect to the factors that discourage surgery as a profession, 45.7% (234/492) of the surgeons provided at least one argument related to the discouraging work characteristics, and 67.6% (346/492) of the surgeons provided problematic contextual characteristics.

**Conclusion:**

This study emphasizes the importance of the calling to surgery as an important factor for choosing surgery as a career. However, the extensive workload, training, and poor work-family balance have been identified as factors that discourage graduates from choosing surgery as a career. The identified positive factors could be used to attract and maintain graduates in surgical disciplines.

## Background

The general demographic development, the limited number of physicians trained in Switzerland, and the changes in the population characteristics of medical students have raised concerns regarding the availability of sufficient physicians, particularly for the near future. Several studies have found that medical students have a declining interest in choosing surgery as a career [1]–[3], especially in Western countries [4], [5].

Previous studies have primarily focused on the perspectives of medical students when investigating the aspects that influence the choice of surgery as a career [4]–[9]. However, students’ decisions regarding their career choices are based on little information, which may be biased by their lack of knowledge of the discipline [10].

One of the main influences on surgery as a career choice, particularly in the early stages of medical training, is the positive impact of role models and mentors [11]–[15]. Thus, surgeons must promote their discipline, and it is important that they understand the concerns that potential candidates have that influence their choice of surgery as a career. It is crucial to increase our understanding of how surgeons perceive the factors that promote or discourage becoming and being a surgeon in today’s graduates. The primary goal of this paper was to identify the factors that surgeons perceive as promoting surgery as attractive or unattractive to today’s graduates.

### Promoting and discouraging factors in career choices

Becoming and being a physician or surgeon is often associated with a calling to the field and discipline [16]–[18]. Several studies have reported a calling to medicine as an important factor in choosing medicine as a career [19], [20], and this is particularly true for surgery [7]. However, a calling for and fascination with surgery are not the only factors that influence career choice. Work psychologists have identified general work and contextual characteristics that are related to work satisfaction, motivation, and job performance across many professions and that influence career choices [21]–[23]. Work characteristics such as meaningful and challenging tasks, being able to use different skills, and having positive social interactions are promoting aspects for a career choice; a demanding workload and a difficult, non-supportive social environment are discouraging factors [24]. In addition, contextual characteristics, and, in particular, threats to a satisfactory work-family balance play major roles in the attractiveness of a career. Many studies have found that both medical students and surgeons perceive surgery as largely incompatible with a satisfactory work-family balance [5], [7], [25]–[28]. Indeed, extensive working hours and work-family imbalance are frequent among surgeons and are the most important contributing factors to surgeon burnout and depression [5], [7], [29], [30]; they are also the major reasons for career changes among residents and attendings [31].

Depending on the surgeon’s experience and the time transpired since medical training, surgeons in different hierarchical situations may perceive different promoting and discouraging aspects with respect to their influence on today’s graduates’ career choices. The secondary goal of this paper was to evaluate whether the perspectives of surgeons in different professional situations converges.

## Methods

In summer 2011, a survey was mailed to 869 board-certified surgeons identified from the membership files of the Swiss Surgical Society as working in Switzerland [32]. To assess the factors that could influence medical students to consider a career in surgery, the following two open-text questions were introduced: (1) “In your opinion, which factors make surgery attractive for today’s graduates?” and (2) “In your opinion, which factors could discourage today’s graduates from choosing surgery as a career option?”. There were no restrictions on the length of the entered text. The survey also contained socio-demographic questions (e.g., age, gender, hierarchical position, hospital category, and language region). The responses were anonymous. The research and ethical committee of Berne, Switzerland determined that this survey did not require ethical approval after it reviewed an outline of the investigation. The data were collected, stored, analyzed, and shared in strict adherence with the ethics committee standards of our institution. The ethics committee did not provide a specific waiver for the present study. Completion of the survey comprised written informed consent to participate in the study. The Swiss Surgical Society database was freely accessible during the time of our study. To ensure the participants’ anonymity, data from the participant questionnaires were entered in an anonymous database.

### Data analysis

The 1,788 written statements provided by the participants were transcribed. Two hundred sixty-three statements did not fit the topic and were not analyzed. The remaining 1,525 statements were analyzed with the following content analysis procedure. First, we defined the level of abstraction for the inductive definition of categories. Second, the content categories were stepwise formulated using an inductive approach; a coding manual was written. Third, each answer was sorted into a final category. Fourth, to determine whether a participant had cited a specific category, we assigned “1” if a category was cited one or more times and “0” if it was not cited. We summarized the subcategories into main categories; a “1” was assigned for the main category if there was at least one category, regardless of how many subcategories were coded.

Cohen’s kappa was used to calculate the inter-rater reliability between two raters. Chi-square tests were used to analyze differences among hierarchical positions. A *P*-value of 0.05 was considered statistically significant, and the tests were 2-tailed. The codings and categorization of the statements were performed with Maxqda, Berlin, Germany [33]. Statistical analyses were performed with SPSS V20.0 software, Chicago, Illinois, USA [34].

## Results

Five hundred twelve of the 869 surgeons submitted the questionnaire (response rate 58.9%). Twenty participants did not specify their position or no longer worked in surgery and were not included in the analysis. The final sample consisted of 492 surgeons: 22 residents (4.5%), 109 (22.2%) attending surgeons, 94 (19.1%) consultants, 123 (25%) heads of departments, and 144 (29.3%) surgeons in private practice. Table 1 summarizes the demographic data of the participants.

**Table 1:** Demographic data of the participants (N = 492).

Characteristic	Value
Hierarchical position	
Resident (board-certified)	22 (4.5)
Attending	109 (22.2)
Consultant	94 (19.1)
Head of department	123 (25)
Surgeon in private practice	144 (29.3)
Hospital category* (2 missing values)	
Type U	68 (13.8)
Type A	116 (23.6)
Type B3	44 (8.9)
Type B2	67 (13.6)
Type B1	41 (8.3)
Private practice	97 (19.7)
Others	57 (11.6)
Language region of the workplace	
German-speaking	376 (76.4)
French-speaking	84 (17.1)
Italian-speaking	14 (2.8)
Romansh-speaking	7 (1.4)
Others (mixed German-French)	11 (2.2)
Women/men	62 (12.6)/430 (87.4)
Age (yr)	49.5±9.6

Values in parentheses are percentages.

*Type U: university hospitals, Type A: large referral centers, Type B3: regional or specialized hospitals, Type B2/B1: small regional surgical. departments.

### Coding accuracy

The inter-rater reliability of the categorizing statements between two coders who independently coded 12.8% (196/1525) of the statements was assessed. Cohen’s kappa was 0.88, which represents high inter-rater reliability [35].

### Results of the content analyses: Promoting and discouraging surgery as a career

The content analyses resulted in three main categories for promoting surgery and two main categories for discouraging surgery as a career. Tables 2 and 3 show the descriptions of the categories, subcategories, and representative examples of the statements for each subcategory.

**Table 2:** Factors that promote the choice of surgery as a career choice.

Main category	Subcategory	Example responses
Task characteristics		
	***Meaningfulness & responsibility:*** Surgery is important, and surgeonshave many responsibilities	“curative”
		“meaningfulness of the profession”
		“responsibility that is very worthwhile”
	***Challenge & task variety:*** Surgery is demanding, includes manydifferent tasks, and is highly dynamic	“challenging job”
		“varied”
		“versatile”
		“dynamic discipline”
	***Teamwork***: Surgery includes collaboration and coordination with otherprofessionals	“teamwork”
		“possibility of teamwork”
Contextual factors		
	***Prestige***: Surgery is a profession with high prestige and is highlyappreciated	“prestige”
		“appreciation”
	Good ***future prospects***: Surgeons have good and many different careerand work opportunities	“bright future prospects ”
		“rather good job opportunities given the anticipated lack of qualified specialists”
	***Regulated working hours***: Regulations limiting working hours forsurgeons are in effect.	“loosen strict requirements concerning working hours”
Surgery as a calling		
	***Fascination***: In general, surgery is interesting and fascinating andallows one to be passionate	“passionate professional”
		“fascination for the subject”
	***Manual skills***: Surgery allows for the use of manual skills	“pleasure of manual activity”
		“handcraft”
	***Skill combination***: Surgery includes a unique combination of manual,intellectual, and social aspects	“handcraft and intellect and humanity”
		“connection hand-heart-brain”
	***Patient care***: Surgery includes close care and contact with patients	“proximity to the patient”
		“contact with patients”
	***Technology:*** Rapid technological progress allows for developingnew techniques and approaches	“technical progress”

**Table 3:** Factors that discourage the choice of surgery as a career choice.

Main category	Subcategory	Example responses
Work characteristics		
	***Extensive workload***: Surgery includes a highly quantitative, physical, andemotional workload	“a lot of night work”
		“physical work load”
		“high stress level”
	***Hierarchy***: Structural aspects and interpersonal relations can be difficult	“hierarchy”
		“arrogance”
		“dealing with colleagues”
		“absolute dependency on mentors and university hospitals”
	***Excessive responsibilities:*** Surgeons carry very high responsibilities	“excessively high responsibility”
		“high responsibility”
	***High demands***: Requirements for becoming and staying a surgeon are very high	“high requirements for candidates”
		“contractual conditions for consultants”
Contextual factors	***Training***: Lack of quality training for prospective surgeons	“long training period”
		“insufficiently structured apprenticeship results in inadequate operative experience”
		“overspecialization“
	***Limited future prospects:*** Surgery as a career has uncertain and unclearperspectives	“uncertainty”
		“lack of prospects”
		“unclear development”
	***Poor work-life balance*** **:** A career in surgery makes it difficult to combinefamily and work	“sacrificing many things (social, family)”
		“raising a family is more difficult for women”
		“working part time hardly possible”
	***Laws and regulations:*** Extraneous regulations by laws and insurancecompanies can be a constraint and limit autonomy	“dependent on politics”
		“health policy”
		“health care insurer”
	***Bureaucracy***: Increasing administrative and bureaucratic requirementsrequire too much time	“little surgery a lot of administration”
		“handcraft is substituted by administration”
	***Loss of prestige:*** Decreasing status, appreciation, and income	“decreasing income”
		“little reward for the effort”
		“less status”
		“it is nothing special anymore”
		“lack of appreciation”

Overall, the surgeons provided more statements that discouraged (945), compared with promoted (580), surgery as a career.

With regard to the promoting aspects, 40.8% (209/492) of the surgeons stated that surgery is a calling, 149/492 (29.1%) provided at least one statement related to the positive task characteristics, and 12.9% (66/492) provided statements related to the supportive contextual factors.

Table 4 summarizes the promoting factors that were identified and separated by each hierarchical level. For all but one category, there were no significant differences among the hierarchical levels. Patient care as a promoting factor was never spontaneously stated by residents, and it was only included by 0.9% of attendings, whereas 8.9% of department heads considered it a promoting factor (*P* = 0.033). Furthermore, 18.2% of the residents described “prestige” as a promoting aspect, while only 3.3% of the department heads did (*P* = 0.059).

**Table 4:** Promoting statements, with the number of participants for categories and subcategories listed by hierarchical position.*

Category	Subcategory	Total number ofparticipants (%)	Resident (n = 22)	Attending (n = 109)	Consultant (n = 94)	Head ofdepartment(n = 123)	Surgeon inprivate practice(n = 144)	CHI2 = (4, n = 492)	P
Task characteristics		149 (29.1)	6 (27.3)	37 (33.9)	30 (31.9)	39 (31.7)	33 (22.9)	4.643	0.326
	Meaningfulness & responsibility	78 (15.2)	1 (4.5)	20 (18.3)	16 (17)	18 (14.6)	22 (15.3)	2.901	0.574
	Challenge & task variety	89 (17.4)	6 (27.3)	22 (20.2)	19 (20.2)	23 (18.7)	16 (11.1)	6.678	0.154
	Teamwork	15 (2.9)	1 (4.5)	4 (3.7)	5 (5.3)	2 (1.6)	2 (1.4)	4.345	0.361
Contextual characteristics		66 (12.9)	5 (22.7)	18 (16.5)	15 (16)	11 (8.9)	16 (11.1)	5.901	0.207
	Prestige	34 (6.6)	4 (18.2)	11 (10.1)	7 (7.4)	4 (3.3)	8 (5.6)	9.072	0.059
	Future prospects	21 (4.1)	1 (4.5)	1 (0.9)	6 (6.4)	7 (5.7)	5 (3.5)	5.041	0.283
	Regulated working hours	16 (3.1)	0 (0)	7 (6.4)	3 (3.2)	3 (2.4)	3 (2.1)	5.105	0.277
Surgery as a calling		209 (40.8)	11 (50)	48 (44)	41 (43.6)	56 (45.5)	47 (32.6)	6.695	0.159
	Fascination	82 (16)	6 (27.3)	18 (16.5)	19 (20.2)	15 (12.2)	20 (13.9)	5.174	0.270
	Manual skills	71 (13.9)	6 (27.3)	21 (19.3)	11 (11.7)	17 (13.8)	15 (10.4)	7.507	0.109
	Skill combination	44 (8.6)	2 (9.1)	5 (4.6)	11 (11.7)	16 (13)	9 (6.3)	7.323	0.120
	Patient care	22 (4.3)	0 (0)	1 (0.9)	5 (5.3)	11 (8.9)	5 (3.5)	10.505	**0.033**
	Technology	36 (7)	1 (4.5)	6 (5.5)	4 (4.3)	12 (9.8)	12 (8.3)	3.433	0.488

*Factors cited in multiple subcategories were counted as one for the respective main category.

With regard to discouraging factors, 45.7% (234/492) of the surgeons provided at least one statement related to discouraging work characteristics, and 67.6% (346/492) described problematic contextual characteristics.

Table 5 summarizes the discouraging factors that were identified and separated by each hierarchical level.

**Table 5:** Discouraging statements, with the number of participants for categories and subcategories listed by hierarchical position.*

Category	Subcategory	Total numberof participants (%)	Resident(n = 22)	Attending(n = 109)	Consultant(n = 94)	Head of department (n = 123)	Surgeon in private practice (n = 144)	CHI2 = (4, n = 492)	p
Work characteristics		234 (45.7)	16 (72.1)	64 (58.7)	36 (38.3)	49 (39.8)	58 (40.3)	19.401	**0.001**
	Extensive workload	193 (37.7)	15 (68.2)	53 (48.6)	32 (34)	40 (32.5)	42 (29.2)	20.699	**0.000**
	Hierarchy	47 (9.2)	6 (27.3)	17 (15.6)	4 (4.3)	6 (4.9)	12 (8.3)	19.673	**0.001**
	Excessive responsibility	21 (4.1)	0 (0)	3 (2.8)	3 (3.2)	4 (3.3)	11 (7.6)	6.175	0.186
	High demands	12 (2.3)	1 (4.5)	2 (1.8)	1 (1.1)	2 (1.6)	4 (2.8)	1.666	0.797
Contextual characteristics		346 (67.6)	11 (50)	68 (62.4)	71 (75.5)	79 (64.2)	105 (72.9)	9.689	**0.046**
	Training	160 (31.3)	10 (45.5)	28 (25.7)	34 (36.2)	45 (36.6)	34 (23.6)	10.266	**0.036**
	Limited future prospects	72 (14.1)	1 (4.5)	19 (17.4)	16 (17)	10 (8.1)	23 (16)	7.386	**0.117**
	Poor work-family balance	74 (14.5)	2 (9.1)	25 (22.9)	14 (14.9)	13 (10.6)	15 (10.4)	10.454	**0.033**
	Laws and regulations	54 (10.5)	1 (4.5)	3 (2.8)	10 (10.6)	17 (13.8)	23 (16)	13.184	**0.010**
	Bureaucracy	59 (11.5)	2 (9.1)	11 (10.1)	7 (7.4)	14 (11.4)	24 (16.7)	5.474	0.242
	Loss of prestige	88 (17.2)	2 (9.1)	20 (18.3)	19 (20.2)	19 (15.4)	26 (18.1)	2.003	0.735

*Factors cited in multiple subcategories were counted as one for the respective main category.

Extensive workload and training issues were most frequently cited as discouraging factors. Residents cited “extensive workload” most often, while surgeons in private practice cited it the least (68.2% vs. 29.2%; *P*<0.001). Residents also cited issues related to training more often than other surgeons (residents 45.5%, surgeons in private practice 23.6%; *P* = 0.036). Other significant differences among surgeons at the different hierarchical levels included concerns with work-life balance (attendings 22.9%, residents 9.1%; *P* = 0.033), concerns with the hierarchical organization (residents 27.3%, consultants 4.3%; *P* = 0.001), and laws and regulatory constraints (surgeons in private practice 16%, attendings 2.8%; *P* = 0.010).

## Discussion

We asked surgeons to consider the perspective of today’s graduates as they consider a career in surgery. This study provides information regarding the promoting and discouraging factors that board-certified surgeons in different stages of their career perceive for entering surgery as a career.

### Surgeons’ perceptions of the factors that promote surgery as a career for today’s graduates

For more than 40% of all surveyed surgeons, surgery as a calling was the most often cited promoting factor for surgery as a career choice; general fascination and the use of manual skills, as well as skill combinations were important subcategories. Becoming and being a physician or a surgeon has often been associated with a calling to the field [16]–[18]. Several studies have reported the calling as an important factor for choosing medicine as a career [19], [20], and this finding holds true for surgery [7]. A recent study showed that 62% of medical students considering surgery as a specialty planned to become surgeons prior to entering medical school; an additional 13% decided during their preclinical training [8]. Personal fit with the job was the most important influence that students cited in planning for a surgical career [7]. The fascination for manual and technical skills that was cited by approximately 14% of the surgeons in our sample reflects the statements medical students use when considering surgery [36].

In our sample, there was only a significant difference in one factor in the category of surgery as a calling among surgeons in different hierarchical levels. The importance of patient care as a promoting factor was significantly more often cited by more advanced surgeons (e.g., consultants and department heads) compared with residents or attendings. This finding may underscore the importance of the manual and technical fascination of surgery as promoting factors; however, it may also reflect a difference in the attitudes of department heads and surgeons at other hierarchical levels.

The second most often cited promoting aspects that surgeons reported were related to task characteristics, such as the meaningfulness of the work, responsibilities, challenging tasks, and task variety. Work psychologists have identified work that is characterized by meaningful and challenging tasks, enables the use different of skills, and provides social interactions is related to high work satisfaction, higher motivation, and better job performance across many professions [21]–[23]. It is important to note that many surgeons in our sample underscored these aspects as potential promoting factors for new candidates for a surgical career.

Contextual characteristics of being a surgeon, such as prestige and future prospects, and the recent regulations of working hours for hospital physicians in Switzerland were cited as positive, albeit less often compared with other aspects. The prestige of the profession was perceived as more important by younger surgeons, whereas department heads and surgeons in private practice considered prestige as somewhat less important for today’s graduates’ career choices. The prestige of the surgical profession has also been cited as a promoting argument in several other studies that investigated the perceptions of medical students, particularly for males [36], [37].

### Surgeons’ perceptions of the factors that discourage surgery as a career for today’s graduates

Overall, surgeons spontaneously provided more statements that described discouraging aspects for a surgical career (580 promoting versus 945 discouraging statements). This finding may be the result of a phenomenon called negativity bias. We recall negative aspects more often and in more detail, and, in general, we pay more attention to negative aspects. However, it may also reflect a more general and critical attitude of surgeons towards their own profession. According to several studies, fewer than half of surgeons would recommend a medical or surgical career to their own children [29], [38], and another study revealed that 18% of surgeons would not go into medicine again [25].

The most often cited specific discouraging aspects were the extensive workload (37.7%), issues of training (31.3%), and problems with work-family balance (14.5%).

Work characteristics such as excessive demands in terms of workload and a difficult, non-supportive social environment are generally stressful across professions [24]. Both extensive working hours and poor work-family balance are among the most consistent findings and the most important factors against choosing surgery as a career. Many studies have found that medical students and surgeons perceive surgery and the work schedules of surgeons as largely incompatible with a satisfactory work-family balance [5], [7], [11], [25]–[28], [36], [39].

Interestingly, in this study, we identified significant differences in the perception of workload and work-family compatibility as discouraging factors among surgeons in different hierarchical positions. Whereas approximately 70% of residents and approximately 50% of attendings spontaneously cited high workload, only one third or fewer consultants, department heads, or surgeons in private practice cited workload as a discouraging factor. Work-family incompatibility was also cited much more frequently by attendings compared with other surgeons. Thus, “older” surgeons (e.g., department heads and surgeons in private practice) appear to clearly underestimate the importance of workload and work-family balance issues for surgery as a career choice. This finding is particularly interesting because the high work load persists during surgical careers and has detrimental effects; high work-load is one of the most important stress factors for surgeons and is related to lower well-being, higher depression, and higher burnout [30], [40]. The same effects hold true for work-family issues. In one study of active surgeons, 50% of surgeons reported at least one important conflict between family and work in the last three weeks, and many conflicts were resolved in favor of work [29]. Similarly, surgeons with poorer work-family balance suffer from higher burnout, a higher prevalence of depression, and less career satisfaction [28], [30], [41]. Work-life imbalance is also one of the main statements that residents and attendants cite for changing their career path to a non-surgical field [31].

One explanation for the differences between the perceptions of more and less experienced surgeons for the two most important factors that discourage a surgical career may lie in the so-called “code among surgeons”. According to Balch and colleagues [28], the self-image of surgeons includes starting work early and finishing late, very long working hours, night- and weekend shifts, and mastering a high volume of work while never complaining. Long work hours and work-family issues may be seen as a sign of dedication and may even be a source of pride, as well as part of the surgeon’s identity. Another explanation, particularly for the current sample, is that a work-hour limitation (50 hours) for residents was introduced in Switzerland in 2005 [42]. Surgeons whose residencies occurred when working hours were substantially longer may have formed a less negative impression based on the comparison of their situation as residents compared with the new graduates who benefit from the working hour limitation.

The second most often cited discouraging aspect was related to training. It is interesting that training to become a surgeon was viewed as a discouraging factor by approximately one-third of the surgeons, albeit not similarly for surgeons of different hierarchical levels. Residents (45.5%), consultants (36.2%), and department heads (36.6%) cited training as a discouraging factor more often compared with attendings (25.7%) or surgeons in private practice (23.6%).

Other research studies have shown that training is of great concern when deciding whether to pursue a career in surgery. The most critical discussion regarding training is related to its duration [7], [11], [25], [37], [43], [44]. As stated by Collins [45], “mastery of surgery can only be attained through extensive and repeated practice accompanied by appropriate feedback”, which takes several years. This requirement is demanding for residents because surgeons must perform a specific number of different surgeries, learn surgical techniques, and acquire anatomical knowledge in addition to working on the ward to complete residency. However, training can also be a burden for senior surgeons because they have to teach residents and perform safe and successful surgeries at the same time.

A further concern for training is the lack of structure in training, which makes it difficult for trainees to plan and ensure they will have an adequate number of cases [6], [25]. It is important to note that the lack of structure and length of training may be connected, and both factors may be related to long working hours. Residency appears to be characterized by a particularly high workload and low work-family balance [36]. Assuring that residents have an adequate number of cases may take substantial time, depending on the case availability during residency training. For this specific aspect, limiting working hours is not advantageous. A previous study showed that regulatory limitations on working hours for residents had a negative effect on training because the limitations to working hours also reduced the time spent in surgery [42].

### Do surgeons make good role models?

Many medical students decide to pursue a career in surgery early in their training [8], [46], and one of the most important influences for entering and staying in a surgical career is the availability of positive role models [7], [14], [15]. Role models are particularly important for students who are undecided but who may be attracted to surgery [27], [47], [48]. Thus, surgeons have a non-negligible influence on recruiting students to surgery. Recent studies have emphasized the importance of early exposure to surgery as an influence that may spark interest, which may also decrease medical students’ negative stereotypes of surgery [14], [46]. Because surgeons may be a crucial influence in promoting surgery as a career, it is important that they know and acknowledge the perspectives of medical graduates on both the promoting and discouraging aspects of surgery. Our study suggests that all surgeons are prone to emphasize positive aspects, such as surgery as a calling and positive task characteristics. However, surgeons higher in the hierarchy may underestimate or even belittle the major concerns of today’s graduates, such as the extensive workload, training issues, or work-family imbalance.

The main strength of this study is that we asked surgeons to take the perspective of a medical student evaluating surgery as a possible career option. This approach enabled the comparison of the students’ perceptions (from previous research) with the perceptions of surgeons at different hierarchical levels, thereby allowing for the identification of convergent and divergent perspectives. Another strength is that the data were derived from open-ended questions. The participants noted the aspects that were most salient to and important for them, and they answered in their own words. Therefore, we obtained a broad and individually weighted spectrum of answers.

A limitation of the study is its geographically limited sample (Swiss surgeons), which does not enable generalization to other areas. Although generalizations to other countries are limited, it is interesting to note the high convergence of the results with other studies. Other limitations include the limited sample size and the unequal participation of surgeons at different hierarchical levels; residents, in particular, were numerically underrepresented.

## Conclusions

The emphasis on surgery as a calling is an important aspect of the survey responses in this study. Although the concept of a career calling cannot be implanted into an individual, i.e., an individual either does or does not have a calling, enthusiasm and dedication from a role model can be contagious and may help undecided students appreciate and weigh the positive aspects of surgery. However, it is also important that interested students see that their concerns regarding training are seen and shared by surgeons, particularly by surgeons who are most involved in training. This acknowledgement of concerns may spark optimism that reflections regarding training issues may produce changes. Another important conclusion of this study is that it is important for surgeons to not underestimate or belittle the most often and most consistent concerns for not pursuing a career in surgery, including the extensive workload and the perceived incompatibility of surgery with having a family. A lack of desire to work in conditions of constant exhaustion and an interest in striving for both a fulfilled work life and family life is not indicative of a lack of dedication; instead, these traits protect against burnout, depression, and health problems. Today’s graduates may not be willing to accept the extensive workload and work-family imbalance as unchangeable components of a surgical career.

## Supporting Information

Survey S1 **Promoting and discouraging factors for choosing a career in surgery.**
(DOCX)
