# Supplementary material for: The Surgeon’s Perspective: Promoting and Discouraging Factors for Choosing a Career in Surgery as Perceived by Surgeons
Source: PLoS One. 2014 Jul 15;9(7):e102756. doi: 10.1371/journal.pone.0102756 (PMC4099181; doi:10.1371/journal.pone.0102756)
Supplement: Survey S1 — Promoting and discouraging factors for choosing a career in surgery. (DOCX) [file pone.0102756.s001.docx]

**Survey S1 Promoting and discouraging factors for choosing a career in surgery**

**1*. In your opinion, what factors make surgery attractive for today’s graduates?***

________________________________________________________________________________________________________________________________________

***2. In your opinion, what factors could discourage today’s graduates from choosing surgery as a career option?***

________________________________________________________________________________________________________________________________________

**Demographic data**

***3. Gender***

1 Male

2 Female

3 Other (if so, please indicate): _______________________________________

***4. Age***

(years)

***5. Hierarchical position***

1 Resident (board-certified)

2 Attending

3 Consultant

4 Head of department

5 Surgeons in private practice

6 Other (if so, please indicate): _______________________________________

***6. Hospital category***

1 U

2 A

3 B3

4 B2

5 B1

6 Private practice

7 Other (if so, please indicate): _______________________________________

***7. Language region of workplace***

1 German-speaking

2 French-speaking

3 Italian-speaking

4 Romansh-speaking

5 Other (mixed German-French)

**Thank you for your help.**
